# Myocardial Viability on Cardiac Magnetic Resonance

**DOI:** 10.5935/abc.20170056

**Published:** 2017-05

**Authors:** Ana Luiza Mansur Souto, Rafael Mansur Souto, Isabella Cristina Resende Teixeira, Marcelo Souto Nacif

**Affiliations:** 1 Universidade Federal Fluminense, Niterói, RJ - Brazil; 2 Centro de Imagem Complexo Hospitalar de Niterói, Niterói, RJ - Brazil; 3 Unidade de Radiologia Clínica - Hospital Vivalle - Rede D´Or - São Luiz, São José dos Campo, SP - Brazil

**Keywords:** Myocardial Infarction / metabolism, Myocardial Revascularization, Tissue Survival, Magnetic Resonance Spectroscopy, Angioplasty

## Abstract

The study of myocardial viability is of great importance in the orientation and management of patients requiring myocardial revascularization or angioplasty. The technique of delayed enhancement (DE) is accurate and has transformed the study of viability into an easy test, not only for the detection of fibrosis but also as a binary test detecting what is viable or not. On DE, fibrosis equal to or greater than 50% of the segmental area is considered as non-viable, whereas that below 50% is considered viable. During the same evaluation, cardiac magnetic resonance (CMR) may also use other techniques for functional and perfusion studies to obtain a global evaluation of ischemic heart disease. This study aims to highlight the current concepts and broadly emphasize the use of CMR as a method that over the last 20 years has become a reference in the detection of infarction and assessment of myocardial viability.

## Introduction

Cardiac magnetic resonance (CMR) has been established as a method to detect myocardial infarction. Using a quick protocol, we are able to obtain information on anatomy, function, tissue characterization, perfusion, and viability, with excellent spatial resolution and image quality. CMR uses different techniques to assess viability, and the technique of delayed enhancement, *per se,* is currently a reference standard for this purpose.

The precise determination of myocardial muscle with or without viability is of extreme importance in the management of a patient with cardiac dysfunction. Viable muscle has a potential for contractile recovery and, therefore, a patient with ischemic cardiomyopathy and ventricular dysfunction may improve his functional capacity after myocardial revascularization^1,2^ and, consequently, have improved survival.^3,4^

The identification of infarcted muscle, even in silent (occult) infarction, is important because this tissue can be a substrate for ventricular tachyarrhythmia,^5,6^ becoming one of the most important causes of sudden death.

CMR presents a state of continuous development, with new tools added at each year to improve this already powerful technology.

This article aims to highlight the current concepts and, in a general way, emphasize the use of CMR as a method that has become over the past 20 years a reference in the detection of myocardial infarction and assessment of viability.

### The pathophysiology of myocardial infarction by CMR

**Figure 1:**
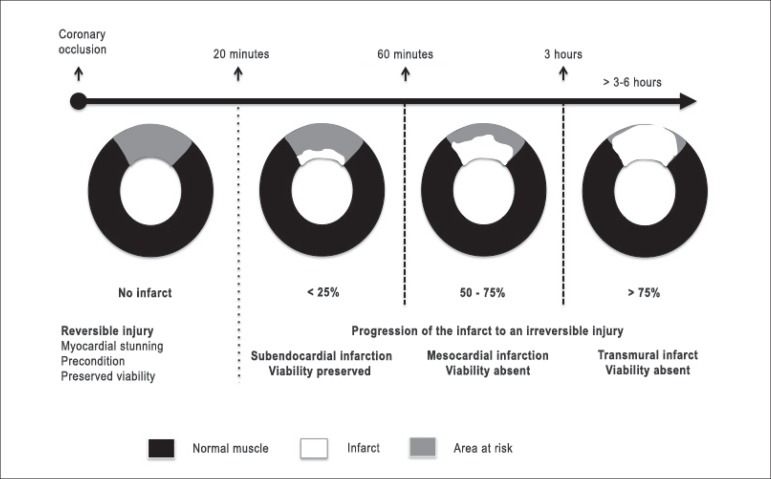
Ischemia duration is the major determinant of the infarct size and its transmural extent. Modified from Arai et al.^7^

CMR's high resolution has transported the concept of transmural extent of infarction from the established theory of experimental studies into the clinical reality.^7^ As illustrated (Figure 1), the duration of the myocardial ischemia is the greatest determinant of the transmural extent of infarction. In a canine model, a coronary occlusion shorter than 20 minutes promotes change in regional contractility without permanent injury or myocardial infarction. The infarction itself develops later and always takes place from the subendocardium to the epicardium. The subendocardium is the most metabolically vulnerable region and one that requires a higher level of oxygenation. After 3 - 6 hours of coronary occlusion, the infarction reaches its transmural extent if reperfusion does not occur.^7^

Other factors can modulate the transmurality of an infarction and its size. Most of these factors are associated with an increased demand of oxygen to the myocyte. In patients with hypertension, tachycardia, or high levels of circulating catecholamines, we can observe an accelerating effect in the establishment of the infarction, causing lesions larger than those in patients without these conditions. In situations involving the level of oxygenation to the myocyte such as anemia, hypoxia, or carbon monoxide poisoning, we will more often also have the establishment of sudden and larger infarctions. In the presence of collaterals, the opposite will occur as a protective effect: the presence of collaterals may reduce the size of the infarction. The same happens when subsequent and short attacks occur in a situation known as precondition (ischemic preconditioning).^8,9^

Drug treatments (beta-blockers), thrombolysis, and/or adequate reperfusion by angioplasty can modify the relationship between the duration of the coronary occlusion and the final size of the infarction. The current problem is that many studies have demonstrated this in animal models, but have failed to demonstrate the same in clinical studies.

We agree with Arai^7^ when he questions the final results of these studies since the reduction of the infarction was measured in the animal model as a fraction of the area at risk. The area at risk is a portion of the myocardium that was hypoperfused during coronary occlusion but did not "die" (infarcted). Some studies have attempted to use sestamibi in the emergency room to assess the area at risk,^10^ but were unable to do so. We consider this outcome as a methodological problem. CMR should be used in this clinical situation, since it does not interfere with the decay of the radiotracer throughout week and time and may, in addition to using the technique of delayed enhancement to quantify the size of the infarction, resort to the T2 technique (edema) in order to assess the area at risk.^11^

In addition to evidence of the existence of some level of myocardial regeneration after acute myocardial infarction,^12^ the final setting after an injury induced by hypoxia in the long term is the replacement of the tissue by fibrosis, defined as replacement fibrosis. In this fibrosis, the area of the infarction is replaced by a scar containing collagen, lacking proteins or structures required for a normal segmental contraction. However, the process of regeneration and the infarct size may influence the possibility of the infarction developing changes in the segmental contractility. In other words, a small myocardial infarction (< 25% of the segment area) probably will not lead to changes in myocardial contractility; on the other hand, a large myocardial infarction (> 75% of the segment area) will promote segmental or regional hypokinesia/akinesia. Thus, the detection of the infarct as present or absent is essential but equally important is the infarct size. Therefore, this replacement and the absence of the tissular actin-myosin mechanism promote loss of contraction and diastolic properties,^13^ which may or may not be macroscopically undetectable by the contractile capacity of neighboring tissues and by the regeneration induced in the segment.

The most important thing is that CMR, with the technique of delayed enhancement, is able to detect myocardial infarction (fibrosis) not detected by clinical evaluation or other methods, such as ECG, echocardiography, and scintiscan.^14,15^ This knowledge is a differential, because even very small areas with delayed positive enhancement, such as approximately 1% of the left ventricular mass, have large prognostic implications.^15-17^

### Myocardial viability

The detection of viable myocardium reflects the presence of living myocytes, and this does not depend on the existence or not of contractile dysfunction or responsiveness of the muscle to external stimuli. Therefore, it is already well established the disconnection between viable myocardium and its pattern of contractility, with several studies demonstrating that the viable muscle could be hypokinetic by chronic cardiomyopathy due to hypoperfusion or acute ischemic cardiomyopathy.^18-20^

The study of myocardial viability is recommended for patients with ischemic cardiomyopathy, and the interest is to know whether a possible revascularization procedure will promote improvement in left ventricular systolic function. In this case, the potential for improvement in contractility will depend on two conditions: first, the muscle must be alive (absence of delayed enhancement); second, the muscle must be ischemic, being this the mechanism of dysfunction. Therefore, myocardial viability is said to be present when a hypokinetic or akinetic muscle features areas without necrosis whose coronary supply is known to be reduced. With this, we imagine that a procedure that restores the blood flow is one that removes the infarct from a hibernating state.^21^

The terms "stunned" and "hibernating" myocardium are used to describe this phenomenon of a condition that is viable, albeit dysfunctional. The reversible phenomenon of the stunned myocardium is identified when the contractile dysfunction develops during acute and intense ischemia, persisting even after restoration of the coronary flow, typically for a period of days to weeks. The reversible phenomenon of the hibernating myocardium, in turn, is identified when the contractile dysfunction takes place during chronic ischemia that is not strong enough to cause cellular necrosis. In this case, the phenomenon fits into the hypothetical concept of pathophysiologic mechanisms capable of inducing adjustment in perfusion-contraction coupling, at a very low level of both, reducing its contractility due to low oxygenation, and avoiding its death.^22-25^

Also noteworthy was the demonstration, as early as 1998, of the CMR ability to detect and quantify the area of microvascular obstruction (no-reflow) associated with an acute myocardial infarction.^26^ The microvascular obstruction is a marker of severe myocardial injury, which is also associated with worse prognosis after acute myocardial infarction.^27^

Unfortunately, the myocardial viability is still highly associated with the detection of segmental or regional contractile dysfunction and is still widely used in the reasoning of clinical cardiologists when they think about the potential of contractile recovery, most likely because the echocardiography is the method most commonly used in clinical practice. With this, we demonstrate in Table 1 the conditions that can cause myocardial dysfunction and are able, in some way, to mask or make difficult a diagnosis of viable myocardium.

**Table 1:** Comparison between conditions that may lead to regional myocardial dysfunction

	Nontransmural Myocardial Infarction	Transmural Myocardial Infarction	Stunned myocardium	Hibernated myocardium	Post-infarction remodeling	Nonischemic cardiomyopathies
Perfusion	Normal or reduced depending on the existence or not of adequate reperfusion or microvascular obstruction	Normal or reduced depending on the existence or not of adequate reperfusion or microvascular obstruction	Normal by definition	Reduced by definition	Normal	Normal
Function	Normal	Reduced	Reduced but reversible with perfusion restoration (hours or weeks)	Reduced but reversible with perfusion restoration (may take months to recover)	Reduced	Normal or reduced(depending on the percentage of the affected area)
Metabolism	Normal or reduced (low FDG uptake)	Reduced (low FDG uptake)	Not reduced (high FDG uptake)	Not reduced (high FDG uptake, perfusion-metabolism mismatch)	Probably normal	Normal or reduced (low FDG uptake)
Histology	Replacement fibrosis	Replacement fibrosis	Normal myocytes	May be normal or present a certain degree of differentiation of the myocytes, with loss and disorganization of cellular elements	Hypertrophy, dilation, and architectural distortion of myocardial fibers	Replacement fibrosis
Delayed Enhancement	Delayed enhancement in the subendocardium (< 50% of the area of the segment). Usually in a coronary territory, unless it has multiple infarctions or the patient has undergone surgery with graft placement	Transmural delayed enhancement that may compromise from the subendocardium to the epicardium (> 50% of the area of the segment). Usually in a coronary territory, unless it has multiple infarctions or the patient has undergone surgery with graft placement	Normal, unless there is a combination of stunned myocardium and myocardial infarction	Normal, unless there is a combination of hibernated myocardium and myocardial infarction	Negative (myocardial dysfunction remote to a large infarction) and, therefore, viable.	Variable, best identified as mesocardial, epicardial, diffuse, or even negative

Table modified from Arai.^7^

**Figure 2:**
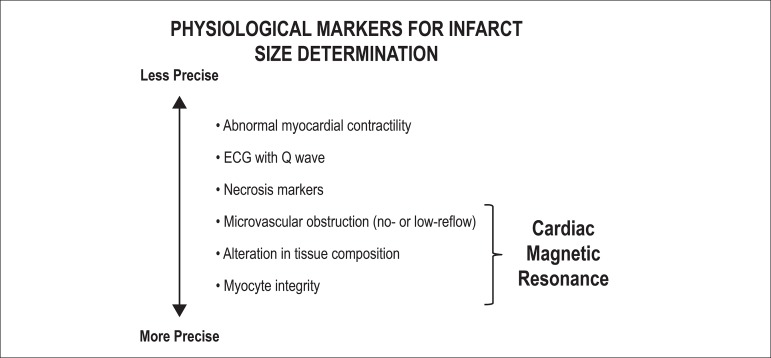
Clinical and physiological markers for determination of the infarct size. Modified from Kaul.^28^

The absence of myocardial viability is the most frequent consequence of a coronary occlusion leading to myocardial infarction. Within this context, a series of parameters may be used to detect whether an infarction indeed occurred and how much of the infarcted territory can be saved. In a review, Kaul^28^ summarized the most accurate markers of infarction, classifying them from less to more precise (Figure 2). For example, the isolated presence of myocardial contractility alteration does not provide information about the presence or absence of infarction, because the hibernating or stunned muscle may be viable but hypokinetic. At the other extreme, the macroscopic and precise identification that magnetic resonance can offer us, with its abilities of tissue characterization, measurement of size, and transmural extent of infarction (delayed enhancement), allows us a better characterization of myocardial viability.

### Technique of magnetic resonance for evaluation of viability

Several techniques of magnetic resonance imaging can be used for the evaluation of myocardial viability, some still without clinical applicability.

- Spectroscopy can be used to evaluate the cellular metabolites and analyze whether the integrity of the myocytes is present or not.^29^ Sodium imaging by magnetic resonance, in turn, may also be a method to differentiate viable from infarcted muscle^30^ and was recently used in volunteers for evaluation of viable muscle with 3-Tesla magnetic resonance. These two techniques have primary limitations in current days due to low signal-to-noise ratio, low spatial resolution, and exceedingly long time for acquisition.
- The use of T1 and T2 images and maps can assist us in the evaluation of myocardial edema and infarction and, in some situations in the areas at risk.^11,31,32^ However, these techniques also have some limitations, the main one related to the fact that changes in T1 or T2 are not specific to detect viability and possible potential for recovery of contractility.
- The use of cine magnetic resonance (cine MRI) can assist in the assessment of segmental and global contractility, or even parietal segmental thickness. These data continue to be of great importance, but there are studies, such as that of Perrone-Filardi et al.,^33^ that have demonstrated that viable muscle may be present in segments with significant parietal thinning. We may also use stressor agents such as dobutamine,^34,35^ adenosine,^36^ regadenoson,^36^ and dipyridamole^37,38^ to assess contractile (cine MRI) or perfusional (first-pass perfusion) alterations, respectively, or even perform combined protocols for multimodal assessment of the myocardium.
- Even with all of the sequences described above, the gold standard in magnetic resonance for the assessment of myocardial viability is the delayed myocardial enhancement study, as discussed below.

### Delayed myocardial enhancement

Studies of delayed myocardial enhancement using T1-weighted magnetic resonance techniques have been reported in the literature since the mid-1980s.^39^ This approach is simple and based on the assumption that infarcted tissue or tissue with increased extracellular space accumulates gadolinium and appears with increased signal on magnetic resonance images (hyperintense signal), mainly in the images acquired 10 minutes after infusion of the contrast medium.

Initially, the shades of gray and white representing the normal and infarcted muscle overlapped due to the inability of the old sequences in detecting lesions with mild to moderate contrast accumulation. In the late 1990s and early 2000s, Kim et al.^40^ and Simonetti et al.^41^ developed a technique able to override gray muscle (normal), highlighting the muscle that accumulates the contrast (infarcted muscle). This technique is currently used on a large scale and is part of all protocols of cardiac examination by magnetic resonance. This has enabled high-resolution images of acute and chronic infarction, demonstrating a high correlation with histopathological studies and virtually equal measurement of myocardial size and viability (myocytes).^40-42^

### Image acquisition and use of intravenous contrast

**Figure 3:**
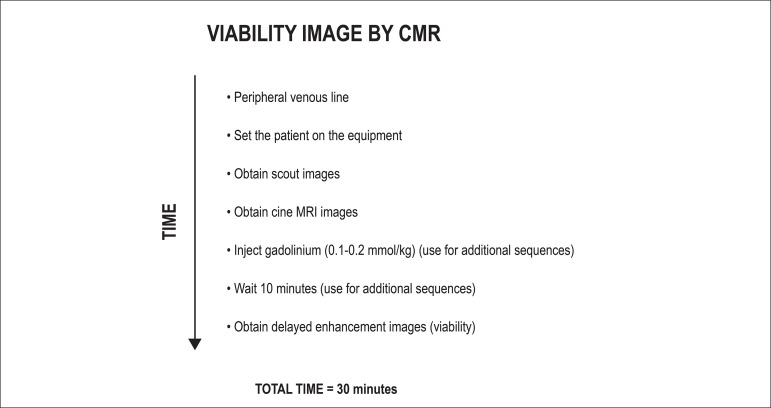
Example of acquisition steps from a protocol for viability/infarction by magnetic resonance. Modified from Weinsaft et al.^20^

The acquisition of delayed contrast-enhanced images is relatively simple and does not require pharmacological or physical stress. Using only a peripheral venous line, we can perform infusion of the intravenous contrast medium gadolinium. After obtaining the scout images, we perform a global and segmental functional study of the right and left ventricle with the cine MRI technique (see specific section). We can, at this moment, infuse gadolinium 0.10 or 0.20 mmol/kg and, after approximately 10 minutes, acquire images of viability (delayed enhancement) of the myocardium in the short, long two-chamber, and outflow axes, as the acquisition of the cine MRI images. Each acquisition of delayed enhancement takes approximately 10 seconds and one apnea, with the entire examination taking an average of 30 minutes (Figure 3).

Delayed enhancement is a technique that can be acquired in different ways, such as 2D, 3D, gradient-echo or inversion-recovery techniques, in addition to those performed in apnea or free breathing.^43-45^ The best technique is chosen and adapted depending on the clinical condition of the patient, the manufacturer of the available equipment, and the experience of the local group.

In practice, an assessment of delayed enhancement (viability) with a short and objective protocol can also be carried out. With this, we are able to abolish the use of cine MRI images and infuse gadolinium alone, acquiring the delayed contrast-enhanced images, which can take only 15 - 20 minutes to be carried out.

Another important factor is the knowledge about the different contrasts used. We currently have approximately 10 different types of gadolinium on the market, and many of them have not been tested for cardiac use, even though they are routinely used.^46^ The most frequently used ones are gadopentetate dimeglumine, gadodiamide, gadoversetamide, and gadoterate meglumine. There is an increasing need for the administration of lower doses of these contrasts to avoid possible adverse effects (Table 2).^46^ In this case, we must always carry out the CMR with the principle of the lowest possible dose in order to establish a diagnosis.

**Table 2:** Gadolinium-based contrasts currently used

Agent	Name	Manufacturer	Recommended dose
Gadopentetate Dimeglumine (Gd-DTPA2)	Magnevist®	Bayer Healthcare	0.1 mmol/kg (0.2 mL/Kg)
Gadodiamide (Gd-DTPA-BMA)	Omniscan®	GE Healthcare	0.1 mmol/kg (0.2 mL/Kg)0.05 mmol/kg (0.1 mL/Kg)§
Gadoversetamide (Gd-DTPA-BMEA)	OptiMark®	Mallinckrodt	0.1 mmol/kg (0.2 mL/Kg)
Gadoteridol	ProHance®	Bracco Diagnostics	0.1 mmol/kg (0.2 mL/Kg)Additional dose of 0.2 mmol/kg (0.4 mL/Kg)
Gadobenate Dimeglumine (Gd-BOPTA)	MultiHance®	Bracco Diagnostics	0.1 mmol/kg (0.2 mL/Kg)
Gadobutrol (Gd-DO3A-butrol)	Gadavist®Gadovist®	Bayer Healthcare	0.1 mmol/kg (0.1 mL/Kg)
Gadofosveset trisodium	Ablavar®	Lantheus Medcl	0.03 mmol/kg (0.12 mL/Kg)
Gadoxetate disodium	Eovist®Primovist®	Bayer Healthcare	0.025 mmol/kg (0.1 mL/Kg)
Gadobenate Dimeglumine (Gd-DTPA- Dimeglumine)	Viewgam®	M.R. Pharma S.A. / Alko do Brasil	0.1 mmol/kg (0.2 mL/Kg)
Gadoterate Meglumine (Gd-HP-DOTA)	Dotarem®Artirem®	Guerbet	0.1 mmol/kg (0.2 mL/Kg)

Other agents have been previously tested but are currently not available in the market: Mangafodipir Trisodium (Teslascan®; GE Healthcare) and Ferumoxides (Feridex®; Amag Pharms). Modified from Nacif et al.^46^

### Quantification of myocardial infarction and prediction of improvement in contractility (viability)

Clinical and animals studies have shown that areas with high signal intensity in the technique of delayed enhancement are highly reproducible when compared with the pathology, especially if the inversion time is properly used. In an animal model, Amado et al.^47^ demonstrated a close correlation between the histopathology and the technique of delayed enhancement (r^2^ = 0.94, p < 0.001). These findings have also been identified with high reproducibility in the clinical setting in acute^48^ and chronic^49^ infarction.

The assessment of viability by magnetic resonance may be performed by a dichotomous approach, strengthened by the Brazilian guidelines.^27^ In accordance with the clinical definition, viability is deemed as present when below 50% of the area of the affected segment, and absent when greater than 50%. We know that this is a categorization of an almost linear phenomenon and that the smaller the necrosis, the greater the probability of improvement in contractility of the segment after revascularization. The reverse is also true, since the greater the necrosis, the smaller the probability of contractile recovery after revascularization.

**Figure 4:**
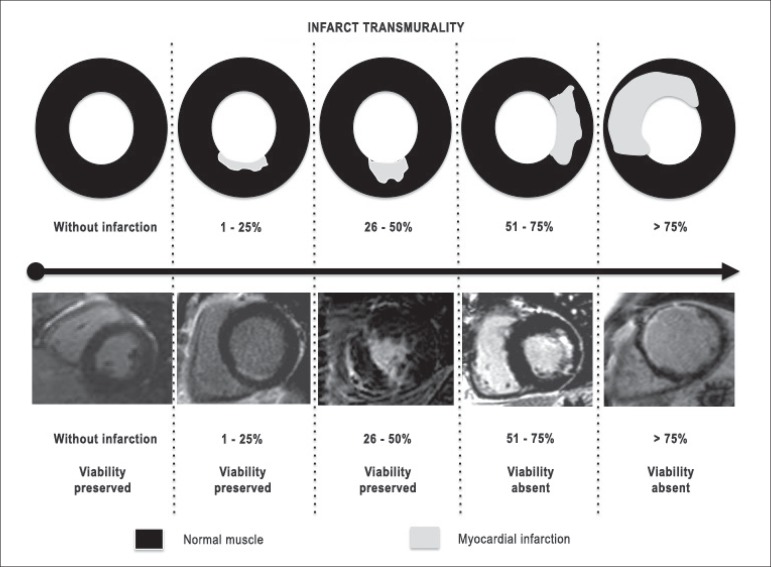
Examples of five different groups of quantification of delayed enhancement, noting that for magnetic resonance, the quantification occurs in a continuum and the potential of viability should be discussed and not just treated as a present or absent dichotomous variable.

In addition to assessment and quantification of fibrosis/global viability, CMR routinely evaluates the potential of contractile recovery in a segmental manner, attempting to characterize the 17 segments of the left ventricle. We divide the groups of quantification of delayed enhancement into five, according to the probability of contractile recovery of the studied segment (Figure 4):^21,50^

- The first is the myocardium without any delayed enhancement, *i.e.*, zero fibrosis/infarction, with a high probability (around 80%) of contractile improvement.
- The second comprises a group with 1 - 25% of the area of the segment with delayed enhancement. In this group, the probability of improvement decreases to 60%.
- The third group includes between 26 - 50% of delayed enhancement, compromising the cardiac muscle, with a probability of improvement in contractility after revascularization of around 40%.
- The fourth group has 51 - 75% of compromised muscle and may present an improvement in contractility in up to 10% of the cases. In this group, we believe that the decision between revascularization and clinical treatment should be widely discussed. The risks of angioplasty or surgery may outweigh the benefits of revascularization, and this depends greatly on the experience and structure of each institution.
- The fifth group comprises those having more than 75% of the area of the myocardial segment compromised, with the potential of contractile recovery of less than 1%.

In addition to this segmental evaluation, we must consider a probabilistic issue related to a certain degree of biological uncertainty, but which we can apply to the 17 segments of the left ventricle. This should be done because the importance will lie in the degree of global systolic functional improvement of the left ventricular ejection fraction. In this way, the improvement in the global function after revascularization may be predicted with great accuracy when considering the threshold of at least 10 viable or normal segments among the 17 segments of the left ventricle according to the classification of the American Heart Association (AHA).^51^

### Medical report of magnetic resonance

The report of a study of myocardial viability must necessarily include the measurement of the left ventricular mass, quantify the area of fibrosis and the transmural extent of infarction, and identify the 17 segments of the left ventricle, according to the studies of Cerqueira et al.,^52^ in order to facilitate the correlation with other imaging methods.

There are several ways to quantify myocardial fibrosis, all of which with scientific value, with some applications being more accurate than others. We encourage the description in the report of the method used to quantify the fibrosis, which may be visual (qualitative)^53^ or by means of a manual software (planimetry),^47^ semiautomatic (with manual correction)^54^ or automatic (without any manual correction).^55,56^

As already well studied and characterized by the MESA (*Multi-Ethnic Study of Atherosclerosis*) study in the work by Rizzi et al.,^57^ several techniques are mostly used to quantify the infarction. We have the visual technique, planimetry (manual), the techniques of standard deviation,^54^ full-width half maximum (FWHM),^58^ and the possible correction of the image noise.^59^ We must remember that all scientific studies exclude images with low technical quality and with artifacts from breathing or acquisition of images, not being possible the analysis by these techniques. Of course, the semiautomatic analysis with manual correction and discard of artifacts becomes the method of choice for quantifying fibrosis and infarction in the day-to-day clinical setting.^56,58,60-68^

**Figure 5:**
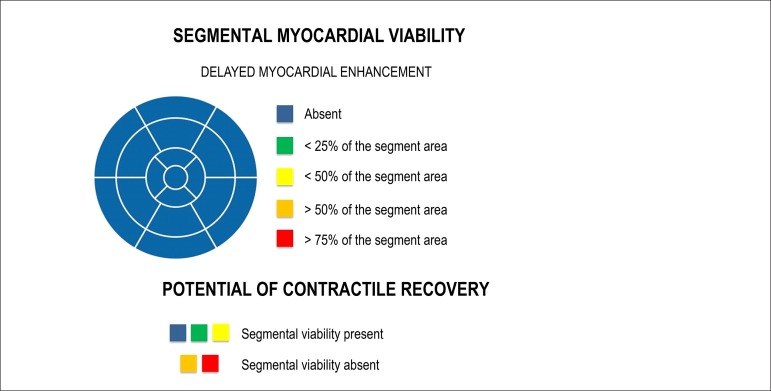
Example of a polar map that can be used in medical reports.

Based on the information described above, we believe that a good way of visualizing such data in the medical reports is the use of polar maps - Bull's Eyes (Figure 5) - to facilitate the explanation of the findings identified by a medical specialist and the understanding of the attending physician.

### Ischemic acute myocardial disease

In the setting of acute illness, rapid myocardial reperfusion prevents the death of viable muscle and improves the ejection fraction and the long-term prognosis.^31,69^ Even after a successful reperfusion, myocardial dysfunction may persist. It is important to distinguish whether this is related to the necrosis or to myocardium stunning. The differentiation between these two clinical situations is of great importance because a stunned muscle must have a significant functional and clinical improvement after coronary reperfusion, either by angioplasty or surgery.^20^

CMR has the ability to differentiate these two clinical situations using the technique of delayed enhancement. This concept was very well studied in the work of Choi et al.,^70^ in which all patients underwent delayed enhancement up to 7 days after the infarction and a second examination between 8 to 12 weeks. In this study, the absence or presence of small foci of delayed enhancement on the analysis of the transmural extent of infarction was able to significantly predict segmental and global functional improvement (p < 0.001). Other studies had very similar results, and we believe that the use of CMR in post-infarction must be routinely encouraged.^71,72^

**Figure 6:**
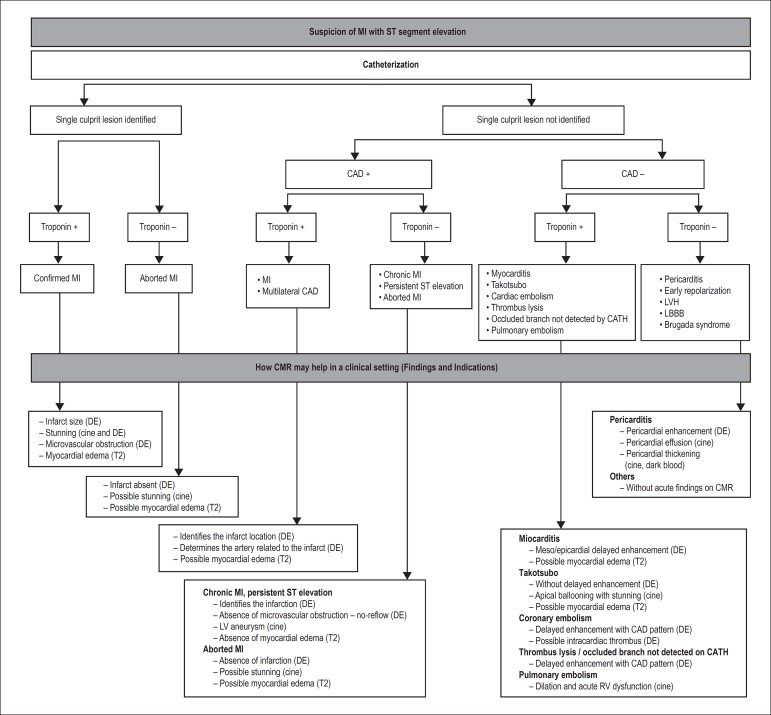
Algorithm for magnetic resonance use in patients with suspected acute myocardial infarction with ST-segment elevation. Based on the presence or absence of signs on cardiac catheterization defining the diagnosis of coronary artery disease and its location and the presence of alterations in markers of myocardial necrosis, the findings from the CMR may lead to a definitive diagnosis of myocardial injury. MI: myocardial infarction; CAD: coronary artery disease; CMR: cardiac magnetic resonance; CATH: catheterization; DE: delayed enhancement; LV: left ventricle; RV: right ventricle. Modified from Kim et al.^50^

In addition to the ability to characterize the infarction, the technique of delayed enhancement is able to modify in practice some diagnosis in our day-to-day clinical setting. As an example, it is relatively common for patients with suspected infarction and normal catheterization to have a definitive etiologic diagnosis demonstrated by CMR, such as myocarditis, vasospasm with reperfusion or, in other cases, small myocardial infarctions caused by occlusion of small vessels undetected in the first analysis on catheterization (in various situations, the report of the catheterization had to be changed after CMR).^21^ In Figure 6, we suggest an algorithm for the use of delayed enhancement in the setting of acute myocardial infarction.

### Chronic ischemic myocardial disease

For over 14 years, magnetic resonance with the technique of delayed enhancement has been used in patients with chronic ischemic disease. The work of Kim et al.^50^ demonstrated the importance of this technique in predicting segmental and global functional improvement after revascularization by surgery or angioplasty.

The transmural extent of infarction is currently of high efficacy to identify those patients who will or will not respond to revascularization. CMR must be used routinely in centers that have this technology.

In a study published by Schvartzman et al.,^73^ the inverse relationship between the transmural extent and functional recovery after revascularization was significant (p < 0.002).

Based on these data, we consider as not being important the use of cutoff values, as currently used, dichotomizing the viability over 50% of the transmural extent as non-viable muscle and results inferior to that as viable muscle. We believe that this is not physiological and may harm some patients who could respond to revascularization even when more than 50% of the transmural extent is affected, since each segment may have an interdependent microcirculation with a certain degree of functional improvement.

### Chronic cardiac failure

When we think of treatment for ischemic disease, we must also include chronic cases of patients with established cardiac failure who depend on the optimization of the clinical treatment.

The use of delayed enhancement and measurement of the transmural extent of infarction has been demonstrated to be a great predictor of response to clinical treatment. In the work by Bello et al.,^74^ magnetic resonance was used in patients with chronic cardiac failure and an ejection fraction of 26 ± 11% before and after 6 months of therapy with beta-blockers. These authors demonstrated improvement in myocardial remodeling and global and segmental function of viable segments.

### Viability and the STICH study

One of the major criticisms in academia regarding the design of the STICH (*Surgical Treatment for Ischemic Heart Failure*) study was the lack of use of magnetic resonance imaging in the identification of myocardial viability since this is a method currently proved to have great reproducibility and increased accuracy. The study randomized patients with ischemic cardiomyopathy who randomly underwent myocardial revascularization or clinical treatment. This scientific design prevents the confusion between medical decision and clinical condition of the patients, and has a great statistical ability to identify the real benefit of choosing between one or other therapy. Unfortunately, even using methods known in the literature such as stress echocardiography and myocardial scintigraphy, the study of viability by these two methods had no ability to identify patients who would benefit from the therapy. Therefore, the STICH study was a negative study for the concept of analysis of viability, which left open the possibility of this hypothesis being tested by magnetic resonance.^21^

The viability by CMR has been tested, and the results were very different from the STICH study. One of the main studies published in the Journal of the American College of Cardiology^75^ showed that the viability, as detected by CMR, was of great importance in differentiating the group with ischemic cardiomyopathy and severe dysfunction of the left ventricle who would benefit from myocardial revascularization. Currently, CMR should be performed in all patients with ischemic cardiomyopathy with left ventricular dysfunction for characterization of myocardial viability.^27^

## Conclusions

Magnetic resonance is able to assess the myocardial viability through a series of different techniques and methods. These techniques can assess metabolic, functional, and morphological alterations and tissue characteristics, in addition to evaluating cellular viability.

The technique most widely used and with the greatest potential for clinical use is delayed myocardial enhancement. This technique is able to identify in a simple and objective way areas of hyperintense signal in the myocardium after administration of the contrast agent, with excellent histologic correlation to characterize areas with infarction/fibrosis.

The technique of delayed enhancement can evaluate myocardial viability not only by a dichotomization between absent and present but also by an almost linear continuum based on the ability of each tissue to recover the contractile capacity.

In addition to the viability, the delayed myocardial enhancement has the ability to detect occult infarcts, characterize lesions that increase markers of myocardial necrosis, and establish an etiological diagnosis of cardiomyopathy, and it may predict an arrhythmogenic potential and risk of death in patients with ischemic or nonischemic myocardiopathy.
